# Systematic review of specialist selection methods with implications for diversity in the medical workforce

**DOI:** 10.1186/s12909-021-02685-w

**Published:** 2021-08-24

**Authors:** Andrew James Amos, Kyungmi Lee, Tarun Sen Gupta, Bunmi S. Malau-Aduli

**Affiliations:** 1 Director of Training in Psychiatry for North Queensland, Queensland Health, Townsville, Australia; 2 College of Medicine and Dentistry, James Cook University, Townsville, Australia; 3 College of Science and Engineering, James Cook University, Cairns, Australia

**Keywords:** Diversity, Justice, Equity, Specialist selection, Residency, Bias, Gender, Ethnicity, Application, Matching

## Abstract

**Purpose:**

There is growing concern that inequities in methods of selection into medical specialties reduce specialist cohort diversity, particularly where measures designed for another purpose are adapted for specialist selection, prioritising reliability over validity. This review examined how empirical measures affect the diversity of specialist selection. The goals were to summarise the groups for which evidence is available, evaluate evidence that measures prioritising reliability over validity contribute to under-representation, and identify novel measures or processes that address under-representation, in order to make recommendations on selection into medical specialties and research required to support diversity.

**Method:**

In 2020–1, the authors implemented a comprehensive search strategy across 4 electronic databases (Medline, PsychINFO, Scopus, ERIC) covering years 2000–2020, supplemented with hand-search of key journals and reference lists from identified studies. Articles were screened using explicit inclusion and exclusion criteria designed to focus on empirical measures used in medical specialty selection decisions.

**Results:**

Thirty-five articles were included from 1344 retrieved from databases and hand-searches. In order of prevalence these papers addressed the under-representation of women (21/35), international medical graduates (10/35), and race/ethnicity (9/35). Apart from well-powered studies of selection into general practice training in the UK, the literature was exploratory, retrospective, and relied upon convenience samples with limited follow-up. There was preliminary evidence that bias in the measures used for selection into training might contribute to under-representation of some groups.

**Conclusions:**

The review did not find convincing evidence that measures prioritising reliability drive under-representation of some groups in medical specialties, although this may be due to limited power analyses. In addition, the review did not identify novel specialist selection methods likely to improve diversity. Nevertheless, significant and divergent efforts are being made to promote the evolution of selection processes that draw on all the diverse qualities required for specialist practice serving diverse populations. More rigorous prospective research across different national frameworks will be needed to clarify whether eliminating or reducing the weighting of reliable pre-selection academic results in selection decisions will increase or decrease diversity, and whether drawing on a broader range of assessments can achieve both reliable and socially desirable outcomes.

## Background

There is long-standing recognition that medical workforces do not represent the diversity of the populations they serve [1]. While there have been improvements in the representation of some under-represented groups, particularly women, as a proportion of medical students and junior doctors, significant imbalances remain among senior doctors and competitive specialties [1–5].

The pattern of under-representation of racial and ethnic minorities is more variable than gender, but equally concerning. One report noted that African Americans, Hispanic Americans, and American Indians comprised more than a quarter of the US population but only 6% of its physicians [1]. The same report argued that increased diversity of the health workforce was justified both to support social justice, and as an effective means of improving population health by improving cultural competence, communication, patient trust, and reducing barriers to care [1, 6]. In response to similar concerns, some medical schools have developed socially accountable education frameworks where community collaboration, equitable selection criteria not solely focused on academic performance, and learning experiences in areas of need are used to encourage recruitment and retention to rural and other underserved populations [7].

Despite the importance of racial and ethnic diversity in the medical workforce there has been less progress in these groups than gender [5, 8, 9]. The barriers to medical workforce diversity are varied, but can be summarised as due to differential resources, selection bias, and anticipated bias [10], leading some to conclude that bias may be reduced if examiners have similar demographics to candidates [11].

A variety of historical and current conditions mean that under-represented minorities (URMs) have fewer material and cultural resources than privileged groups to match the challenges associated with preparing for application to medical school, and for navigating the pathways through medical training to specialist practice [1]. Although it has been argued for some time that the focus on academic performance ignores many of the qualities which contribute to competent, caring, and ethical medical practice [12], there has been little progress in developing and implementing reliable non-academic indicators of aptitude for medical practice [13]. As Roberts et al. [14] make clear, all current methods of selection into medical specialty training may contribute to biased selection. The most reliable instruments used for selection into medical specialties are multiple choice question (MCQ) tests, because the format allows for a large number of items and a broad coverage of content. Efforts to improve the validity of selection decisions are less well developed, although there has been an effort in the UK to improve the validity of selection decisions by developing a suite of reliable measures across a range of relevant skills and knowledge.

Biased measures during trainee selection may be one cause of under-representation of some groups in medical specialties, tending to favour privileged groups [14]. For example, men have shown a small but reliable advantage over women on the MCQ tests used for medical school selection, while women have shown an advantage on the clinical assessments performed during medical school [15]. Perhaps anticipating this type of selection bias, or as a result of differential resources, URMs may be less likely to apply for medical school or specialist training than other people with similar levels of ability [16].

### The broader medical training selection literature

Useful context is provided by two recent reviews which describe a tension between the reliability and validity of the processes and instruments used for selection along the training trajectory from medical school through to consultant practice. After canvassing the significantly different trajectories in different countries through medical school, selection into generalist training, and transition to consultant practice, Roberts et al. [14] propose two basic national patterns of medical specialty training selection (MSTS) with the US representative of a pattern of relatively greater dependence upon pre-selection academic achievement combined at the local level with subjective measures such as letters of recommendation; and the UK in the early stages of developing a systematic framework that combines multiple reliable methods of selection covering a broad range of skills.

The heavy reliance of the US MSTS framework on pre-selection academic achievement is illustrated by the status of the United States Medical Licensing Exam - Part I (USMLE I) as the most common tool used for MSTS in the US, despite being created for licensure as a doctor at the end of medical school [14]. The USMLE I is very attractive to administrators responsible for MSTS decisions because of its convenience as a reliable, standardised, pre-existing measure allowing the direct comparison of a large majority of US doctors on a measure of characteristics ostensibly relevant to specialist practice without the need for additional testing. These benefits are so significant that they overwhelm the questionable validity of using the same test to select into specialties as diverse as psychiatry, surgery, and paediatrics, and in fact have been argued to have prevented the development of more valid measures targeting specific specialties [17].

This tension between reliability and validity, with the strong temptation to focus on reliability for its administrative convenience, is an example of the long-recognised problem that focusing management only on what is most conveniently measured ignores crucial factors which may not be so easily measured [13, 18]. Social accountability theory suggests that selecting candidates for entry into medical school or medical specialties based purely on pre-selection academic achievement is likely to ignore many socially important goals, often exacerbating existing inequities [19].

Due to the overlapping methods and analysis, and the larger dataset, further context is available from Patterson et al’s [20] review of the methods of selection into medical school. They conclude that the validity and reliability of selection decisions may be improved by developing specific measures using structured techniques such as situational judgement tests (SJTs) and multiple-mini interviews (MMIs) (both described in Table 1), while the greater reliability of pre-selection academic achievement measures may involve the cost of preventing the entry of some under-represented minorities into medical training. Both these reviews illustrate the over-reliance of medical selection research on retrospective, cross-sectional designs and the tendency to focus on reliable more than valid indicators. While a full exploration is beyond the scope of this review it is useful to note that the tension between reliability and validity is important outside the boundaries of academic medicine. The large size and crucial social functions played by health workforces makes their composition a live political issue, leading to calls for the reduction of the reliance on standardised tests to improve the diversity of selection into health professions more generally, which may be interpreted as a restatement of the tension between reliability and validity translated into more commonly understood language [1, 21].

**Table 1:** Common instruments for selection into medical specialist training programmes [14, 20]

Instrument	Description
*Interviews/Multiple mini-interviews*	Includes standardised and non-standardised interviews, which may be supported by psychometric evidence, although frequently involve subjective judgements.
*Academic records*	Particularly school results measured against a year-cohort, but may include other information, such as extra-curricular activities, awards, etc
*Standardised exams/aptitude tests (including SJT/CPST)*	Includes exams which test general medical, not specialist, aptitude: • Standardised exams used for selection into medical school or licensure for practice, such as the United States Medical Licensing Exam(s) and the UK’s Multi-Specialty Recruitment Assessment And exams designed for particular specialties, including: • OSCE format interviews • Situational judgement tests which assess non-cognitive characteristics by presenting workplace-based scenarios requiring non-clinical decisions • Clinical problem-solving tests (CPST) which involve multiple-choice responses to clinical scenarios requiring clinical reasoning
*Curriculum vitae*	Structured or free-form document(s) provided by candidate outlining their education, training, and work experiences.
*Letters of recommendation*	Structured or free-form letters expressing an opinion on the candidates’ specific or general capacities, often weighted for the perceived expertise or prestige of the undersigned; for example greater weight may be given to a LoR by the Dean of a prominent medical school than a consultant in a medical specialty.
*Personal statements*	Structured or free-form statements by the candidate usually addressing specific criteria such as motivation, priorities, and personal circumstances.
*Referees reports/references*	Structured or free-form reports by referees with knowledge of the candidate addressing specific selection criteria.
*Locally defined criteria*	The criteria used for selection into individual specialist training programmes may not be precisely defined. Locally defined criteria may involve algorithms weighting various of the instruments described above, and may or may not involve objective thresholds or subjective judgements

### Review goals

In the context of the tension between the reliability and validity of MSTS measures and the pragmatic advantages of reliable measures, this article was designed to review and evaluate the research on how MSTS instruments affect the diversity of selection into medical specialty training programs, and make recommendations for balancing the goals of reliable and equitable MSTS, justifying the following research questions:

- What URMs have been considered regarding the impact of empirical MSTS methods on diversity?
- What research designs have been used to examine the impact of empirical MSTS methods on diversity?
- What evidence suggests that reliance on measures of pre-selection academic achievement decrease MSTS diversity?
- What evidence suggests that novel selection processes improve diversity relative to pre-selection academic achievement measures and what is their impact on reliability?

## Method

### Study selection

Study inclusion/exclusion criteria are presented in Table 2. To focus on the effect of specific measures used in the decision to accept candidates into specialty training, studies which reported surveys or other ways of measuring candidate perceptions, motivations, and preferences were excluded. Table 1 describes the common instruments used for selection in the literature.

**Table 2:** Inclusion and exclusion study criteria

Inclusion criteria	Exclusion criteria
• Selection into medical specialty training program • Results report empirical evidence about a measure used for medical specialty selection • Focus of article is on diversity or under-represented minority in medical specialty training • Published between 1.01.2000 and 31.12.2020 • English	• Selection into medical school • Selection into non-medical training: ○ Nursing ○ Allied Health ○ Dental ○ Pharmacy • Articles where diversity or underrepresented minority in medical specialty training is not the focus • Not in English • Published prior to 1.1.2000 or after 31.12.2020 • Survey results only • Empirical results relate only to preferences, perceptions, motivations to apply, and not measures used as basis of selection

### Search strategy

The search was based on the method suggested by Aveyard [22]. Searches were repeated in PubMed/Medline, PsycINFO, Scopus, and ERIC, in order to identify relevant articles from the medical, psychological, and educational literature (see search strings in Supplementary materials). Search results were supplemented with hand-search of key journals, articles in the reference lists of the articles selected for inclusion in the review, and articles which cited the articles selected for inclusion in the review (identified using Web of Science). Key journals were defined as those with two or more articles selected for review, including: Medical Education, BMC Medical Education, and Academic Medicine.

During the search, the terms used for doctors in medical specialty training included “resident”, “trainee”, and “postgraduate”. Where specific instrument or minority search terms were added to the basic search, they were added as “OR” clauses that would return a larger set, and never used to constrain/reduce searches. Such additional search terms referred to specific instruments of selection used in the US (United States Medical Licensing Exam – USMLE; of several parts USMLE 1 and USMLE 2 are commonly used for selection) and the UK (SJT – Situational Judgement Test, CPST – Clinical Problem Solving Test). The two most common URMs, gender and international medical graduates, were also specifically added. A broad net was cast for articles about diversity including the terms divers*, equit*, gender, foreign, international, underrepresented, and minority.

### Data extraction and analysis

Each article was reviewed with reference to a standard data extraction pro-forma designed for this study (see Supplementary materials). An excel spreadsheet collected and summarised information from the pro-forma. Methodological strengths and limitations were systematically collected and coded in relation to scope of study, research quality, sample size, power analysis, specialty and length of study/ follow-up.

We used the Medical Education Research Study Quality Instrument (MERSQI) as a standardised measure of article quality [23, 24]. This instrument covers six domains comprising study design, sampling, type of data, validity of evaluation instrument, data analysis, and outcomes measured, with scores varying between 5 and 18. Two of us (AA & BMA) independently completed the MERSQI for each article, and resolved disagreements with reference to MERSQI criteria in a joint session, achieving consensus. A recent review of studies using the MERSQI to assess the quality of medical education studies reported a range of overall scores between 8.9–15.1 (max 18) with a median of 11.3, while recommending that quality should also be assessed by examination of the specific features and conditions of individual studies.

### Post-hoc analysis of unbalanced results

In response to the search results, with a single article (from Canada) outside the dominant set from the US and a smaller set from the UK, it was decided to analyse what impact the use of specific search terms including instruments used primarily in the US (USMLE) and UK (SJT/CPST) and specific minority groups (gender/IMG) had on the search results. As we used specific terms only to increase the number of hits and not to decrease them, we do not think it was possible to have introduced a bias against finding research with particular characteristics (such as research done outside the US/UK). However, it seems possible that using specific search terms could have misrepresented the literature by tending to return a greater proportion of US/UK and/or gender/IMG articles. We tested this in two ways: to examine whether we might have missed additional articles eg from other countries, we extended our search over the 2000–2020 time period, to a fourth database, Scopus, the largest database available to us. To quantify the potential bias of having a greater probability of identifying articles from US/UK than elsewhere we identified the articles which were included in our review which were not identified by our basic search, but which were added as a result of the specific search terms above.

## Results

**Fig. 1:**
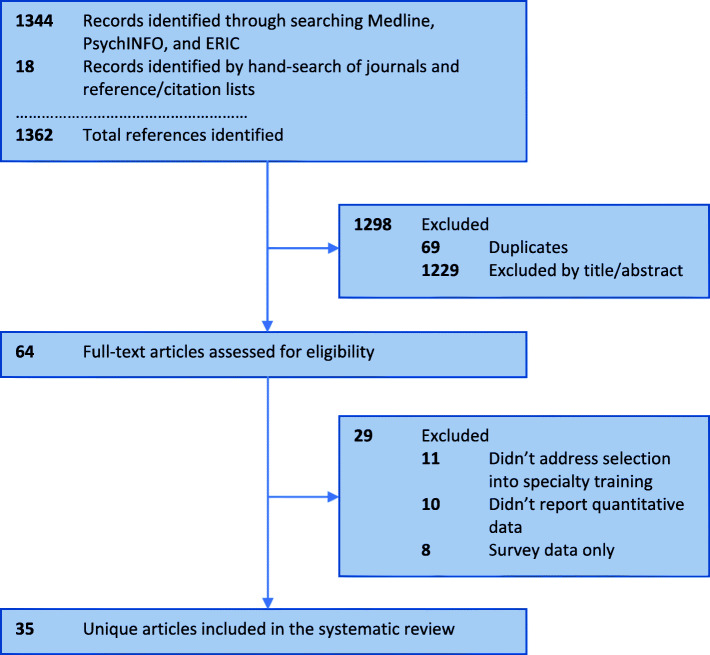
PRISMA Flowchart of literature search and article inclusion/exclusion

The database searches retrieved a total of 1344 abstracts with 1275 unique articles after 69 duplicates were removed (Fig. 1). Eighteen articles were added after the hand-search of key journals and reference/citation review. Application of the inclusion/exclusion criteria identified 64 articles for full-text retrieval, and full-text review yielded 35 articles for inclusion in the article.

The retrieved articles comprise a heterogeneous set with few commonalities, described below and summarised in Table 3.

**Table 3:** Summary of Reviewed Articles

Article (bolded authors claimed evidence of bias)	Description	Main findings	Diversity conclusions	Strengths/limitations	MERSQI Score^**a**^ (11.3/18 over all articles)
*Canada (1 article)*					*9*
MacLellan et al. (2010) [25]	Compared IMG and DMG performance on in- and end-training exams	End-training exam pass rate: IMG 56% versus DMG 93.5% (*p* < .0001)	**IMG:** IMG low pre-selection scores consistent with low pass rates on certification exams	**Strengths:** Multiple year, large sample **Limitations:** Exploratory, single program, single specialty	**9**
*UK (7 articles)*					*15.2*
**Esmail et al. (2013)** [26]	Compared IMG with DMG performance on end-training exams (GP/Family medicine)	URM failed first attempt more than white DMG (OR 3.5, *p* < .001) IMG failed first attempt more than white DMG (OR 14.7, *p* < .001)	**URM/IMG:** Higher failure rates in domestic and foreign URM/IMG are partly explained by lower pre-selection academic achievement, and may also reflect bias during clinical OSCE-based exams	**Strengths:** Complete cohort, large sample, multiple years, end-training outcome **Limitations:** Exploratory, single specialty	**15.8**
McManus et al. (2014) [27]	Compared IMG with DMG performance on end-training exams (GP/Family medicine & Internal medicine)	IMG performed worse than DMG on end-training exams (~ 1.25 SD)	**IMG:** Lower pre-selection scores are an accurate measure of suitability for training Raising cutoffs is needed for equivalence with DMG but would affect workforce	**Strengths:** Follow-up study, multiple programs, large sample, multiple years **Limitations:** Two specialties	**15.2**
**Patterson et al. (2018)** [28]	Measured factors associated with differences in performance of IMG and DMG on end-training exams (GP/Family medicine)	Clinical skill performance better predicted by SJT than CPST (beta 0.26 v 0.17) SJT mediated relationship between English fluency and clinical skills performance	**IMG:** IMG performance on end-of-training exams is predicted by socio-linguistic factors not clinical knowledge and skills	**Strengths:** National cohort, large sample, multiple years, end-of-training follow-up **Limitations:** Exploratory study, single specialty	**14.6**
Tiffin et al. (2014) [29]	Measure IMG performance during residency	IMG more likely to receive unsatisfactory ARCP than DMG (OR 1.63, *p* < .05)	**IMG:** PLAB language exam does not establish linguistic equivalence of IMG and DMG Thresholds would need to be increased to achieve equivalence, but would affect workforce and decrease diversity	**Strengths:** National cohort, large sample **Limitations:**	**14.6**
**Tiffin et al. (2018)** [30]	Measure bias against IMG in resident selection comparing pre-training academic attainment with in-training assessment	UK overseas graduates more likely deemed appointable than IMG (OR 1.29, *p* < .05) but more likely to later receive less satisfactory ARCP (OR 1.20, *p* < .05)	**IMG:** Bias favouring UK born graduates trained overseas versus IMGs may be due to excessive weight given to interview	**Strengths:** National cohort, large sample, all specialties, **Limitations:** Incomplete data set	**15.8**
Wakeford et al. (2015) [31]	Measure correlation between GP/Family medicine and Internal medicine exam performance by ethnicity	High correlation between GP/IM exam performance, suggesting validity of each assessment (and does not suggest bias against URM) URM performed less well	**URM:** No evidence of bias against URM; differences in assessment likely to reflect true differences in ability	**Strengths:** National cohort, multiple years, large sample **Limitations:** Exploratory, two specialties	**15.8**
Woolf et al. (2019) [32] *Identified by specific search terms*	Measure effect of gender on specialty training selection	Across all specialties female applicants had: • No difference in applications • Increased offers (OR 1.4, *p* < .001) • Increased acceptance (OR 1.43, *p* < .001) 2 specialties had significant gender differences in applications (both favouring women): • Paediatrics (OR 1.57, *p* < .05) • GP (OR 1.23, *p* < .05)	**Gender:** Gender segregation in specialties is due to differential application rates, not instrument bias; research is needed on why men are less likely to apply for GP/Paediatric training, and less likely to accept GP training if offered	**Strengths:** Follow-up study, national cohort, large sample, multiple specialties **Limitations:** 1–2 years intake, incomplete data set	**14.6**
*US (27 articles)*					*10.4*
Aisen et al. (2018) [33] *Identified by specific search terms*	Examine effect of gender on **urology** applicant academic achievement and selection into specialty	Higher % of males matched (73% v 67%) Among matched applicants: • Males less honors (2.8 v 2.2, *p* < .021) • Males higher USMLE1 (245.9 v 240.8, *p* < .001)	**Gender:** Male/Female candidates had similar pre-selection results and no evidence of bias in selection	**Strengths:** Moderate size **Limitations:** Exploratory, single program, single specialty, 1–2 years intake	**11.3**
Brandt et al. (2013) [34]	Examine effect of gender on **O&G** applicant academic achievements and selection into specialty	No gender difference on USMLE Females more likely to have honors (51% v 41%, *p* < .021) and published (87% v 79%, *p* < .01)	**Gender:** Male/Female candidates had similar USMLE1 scores, higher female honors may explain lower rate of M applications for O&G training	**Strengths:** Large sample, multiple years **Limitations:** Exploratory, single program, single specialty, incomplete data set	**11.3**
Chapman et al. (2019) [35]	Identify factors associated with under-representation of women across **medical specialties**	Female representation higher in specialties with lower mean USMLE1 entry score (*p* < .017) 1% increase in female faculty prevalence associated with 1.45% increase in female trainees in specialty (*p* < .001)	**Gender:** No evidence of USMLE 1 bias against females Association between female faculty and female trainees suggests mentoring may increase diversity	**Strengths:** National cohort, large sample, all specialties **Limitations:** Exploratory, 1–2 years intake, incomplete data set	**9**
**De Oliveira et al. (2012)** [36] *Identified by specific search terms*	Measure factors associated with selection to **anaesthetics** residency including gender, age, country of training	Factors associated with selection: • Female • Younger • Higher USMLE 2 • DMG	**Gender/Age:** Bias favouring selection of **female** and **younger** applicants	**Strengths:** Large sample **Limitations:** Exploratory, single program, single specialty, 1–2 years intake, inferences made without statistical test	**12.4**
Dirschl et al. (2006) [37] *Identified by specific search terms*	Measure whether gender and academic scores can predict **orthopaedic** end-of-training exams	12.5% female applicants Faculty ratings of training were not associated with academic scores	**Gender:** No gender bias detected	**Strengths:** Follow-up study, large sample, multiple years **Limitations:** Single program, single specialty	**9**
Driver et al. (2014) [38]	Identify factors associated with **ophthalmology** selection including IMG status	Increased % of selection associated with: • Higher USMLE1 (OR 3.22, *p* < .05) • Letters of recommendation (OR 6.2, *p* < .05) • Publications (OR 3, *p* < .05)	**IMG:** Design prevented conclusions about bias	**Strengths:** National cohort, large sample, multiple years **Limitations:** Exploratory, single specialty	**11.3**
**Durham et al. (2018)** [39]	Measure effect of gender on selection into **neurosurgical** training	13.8% female applicants USMLE1 higher for selected (233 v 211, *p* < .001) Females had lower OR of matching (0.59, *p* < .001) Females had lower mean USMLE1 scores (222 v 230, *p* < .001)	**Gender:** USMLE 1 is best predictor of selection **Reduced female** selection partially explained by lower USMLE 1 scores Possible bias remains after multivariate analysis	**Strengths:** Statewide cohort, large sample, multiple years **Limitations:** Exploratory, single specialty	**11.3**
**Edmond et al. (2001)** [40] *Identified by specific search terms*	Measure bias against African Americans due to **USMLE 1** in **internal medicine** residency selection	Mean USMLE1 of African Americans was 200, non-AA was 216 OR for rejection of AA varied from 3 to 6 (*p* < .05)	**Race:** USMLE 1 reduces selection of **African Americans**	**Strengths:** Large sample **Limitations:** Exploratory, single program, single specialty, 1–2 years intake, uncontrolled confound	**12.4**
**Filippou et al. (2019)** [41]	Measure gender bias in letters of recommendation for **urology** resident applicants	LoR for males had: • More authentic tone • More references to personal drive, work, and power LoR referring to power more likely to be associated with selection	**Gender:** Gender bias in letters of recommendation may reduce selection of **females**	**Strengths:** Moderate sample **Limitations:** Exploratory, single program, single specialty, 1–2 years intake	**9**
French et al. (2019) [42]	Measure gender bias in LoR for **general surgery** resident applicants	Female authors wrote longer letters	**Gender:** No gender bias detected in letters of recommendation	**Strengths:** Large sample, adequate power **Limitations:** Exploratory, single program, single specialty, 1–2 years intake	**7.9**
**Friedman et al. (2017)** [43]	Measure gender bias in standardised versus narrative LoR for **otolaryngology surgery** residents	No difference in ranking of male/female applicants Female writers produce LoRs different to male writers (p < .05) LoRs written for female applicants less positive than those written for male applicants (*p* < .05)	**Gender:** Standardised letters of recommendation have reduced but not eliminated biases that contribute to reduced selection of **females**	**Strengths:** Moderate sample **Limitations:** Exploratory, single program, single specialty, 1–2 years intake	**7.9**
Gardner et al. (2019) [44]	Measure effect of USMLE cutoffs on underrepresented minorities in **general surgery** training	Reducing USMLE1 cutoffs and adding SJT screening increased URMs offered interview by 8%	**Gender/URM:** USMLE 1 screening reduces selection of URMs for interview Does not claim bias	**Strengths:** Multiple program sample, large sample **Limitations:** Exploratory, single specialty, 1–2 years intake	**9**
Girzadas et al. (2004) [45]	Measure effect of gender on SLoR for **emergency medicine** residency	Female author with female applicant OR 2 to get highest ranking on LoR (*p* = .023)	**Gender:** No gender bias detected in letters of recommendation	**Strengths:** Large sample **Limitations:** Exploratory, single program, single specialty, 1–2 years intake, selection process changed during study	**7.9**
**Hewett et al. (2016)** [16]	Measure gender bias in **radiology** residency selection	24% female applicants Females were • 30% of offered interviews • 38% of top quartile (*p* < .001) • 25% of selected Female applicants average USMLE1 score was 5 points lower (*p* < .05) Female applicants had higher mean interview scores (*p* < .05)	**Gender:** Bias **favouring female** applicants Associated with lower female USMLE1 scores Associated with higher female interview scores	**Strengths:** Multiple years intake, large sample **Limitations:** Exploratory, single program, single specialty, variable selection/scoring methods	**11.3**
**Hoffman et al. (2020)** [46]	Measure gender bias in LoR for **pediatric surgery** residency selection	Female LoR had more communal phrases (*p* < .01)	**Gender:** Gender biases **against females** in LoRs may affect selection into training	**Strengths:** Multiple years intake **Limitations:** Exploratory, single program, single specialty, small sample, ad-hoc measures	**7.9**
**Hoffman et al. (2019)** [47]	Measure gender bias in LoR for **transplant surgery** resident applicants	Male applicant LoR had more agentic terms (*p* < .05) LoR written by senior staff more likely to describe female applicants with communal terms (*p* < .05)	**Gender:** Gender biases in LoRs **against females** may affect selection into training	**Strengths:** Moderate sample size, multiple years intake **Limitations:** Exploratory study, single program, single specialty, limited power	**7.9**
Hopson et al. (2019) [48] *Identified by specific search terms*	Measure influence of gender on outcome of **emergency medicine** selection interviews	No significant difference on standardised video interview	**Gender:** No gender bias detected on standardised video interview	**Strengths:** Multiple program cohort, large sample size, adequate power reported **Limitations:** Exploratory study, single specialty, 1–2 years intake, aggregates heterogenous groups, ad-hoc measures	**10.1**
Kobayashi et al. (2019) [49]	Measure influence of gender on LoR in **orthopaedic** surgery residency	Female applicants had: • Longer LoR (*p* < .003) • More “achieve” words (*p* < .0001) No differences for male v female authors	**Gender:** No gender bias detected on letters of recommendation	**Strengths:** Large sample **Limitations:** Exploratory study, single program, single specialty, 1–2 years intake, ad-hoc measures	**11.3**
**Lin et al. (2019)** [50]	Measure gender bias in LoR for **ophthalmology** residency	M/F applicants had similar: • USMLE1 • Academic achievement LoR for male applicants had: • Less feel words (*p* < 041) • Less biological words (*p* < .028)	**Gender:** Gender biases in LoRs **against females** may affect selection into training	**Strengths:** Moderate sample size **Limitations:** Exploratory, single program, single specialty, 1–2 years intake, ad-hoc measures	**11.3**
**Lypson et al. (2010)** [51] *Identified by specific search terms*	Measure correlation between USMLE scores and clinical competence at beginning of residency **across specialties**	USMLE1 scores lower for URM (212 v 230, *p* < .001) URM not significantly worse than non-URM on OSCE stations at beginning of residency	**URM:** USMLE 1 scores are biased against URMs, revealed by similar OSCE scores at beginning of residency	**Strengths:** Multiple specialties, multiple years intake **Limitations:** Exploratory, single program, small sample, limited power	**7.9**
Norcini et al. (2014) [52]	Predict patient outcomes of IMGs from USMLE scores **across specialties**	Increased USMLE2 CK score associated with decreased mortality as a physician 1 SD on USMLE 2 CK associated with 4% improvement in mortality	**IMG:** USMLE2 CK scores are a valid measure of suitability for IMG selection/certification	**Strengths:** Follow-up study, statewide sample, large sample, multiple specialties, multiple years intake, patient outcomes **Limitations:** Unmeasured confounds	**14.5**
Poon et al. (2019) [53] *Identified by specific search terms*	Compare **orthopaedic** residency enrolment rates and academic metrics of applicants and matriculated residents by race/ethnicity	URM were 29% of applicants and 25% of enrolments White/Asian applicants had higher USMLE1 than Black applicants (234 v 218, *p* < .05)	**URM:** USMLE1 screening may contribute to lower rates of application of URMs Bias not evaluated	**Strengths:** National cohort, large sample, adequate power **Limitations:** Important variables not measured	**13.5**
**Quintero et al. (2009)** [54]	Measure effect of personality similarity to bias the selection of **orthopaedic** residents	Clinicians rated candidates more favourably when they shared personality characteristics (*p* = .044)	**Personality:** Increased awareness of implicit biases may reduce inequity of current selection processes	**Strengths:** Moderate sample size **Limitations:** Exploratory, single program, single specialty, 1–2 years intake, limited power, follow-up to selection, protocol variations	**12.4**
Scherl et al. (2001) [55]	Measure gender bias in **orthopaedic** resident selection	No significant difference in selection of male and female charts	**Gender:** No gender bias detected based on gendered versions of applicant charts	**Strengths:** Experimental design **Limitations:** Exploratory, single program, small sample, selection bias, partial blinding	**11.3**
Stain et al. (2013) [56] *Identified by specific search terms*	Measure attributes of top-ranked applicants to **general surgery** residency	Males had higher USMLE1 (238 v 230, *p* < .001) Males/Females had similar USMLE2 scores (245 v 244, *p* = .54) Highly competitive programs associated with • USMLE1 (RR 1.36) • Publications (RR 2.2) • Asian (RR 1.7 v white)	**Gender:** No gender bias detected based on pre-selection academic achievements	**Strengths:** National cohort, moderate sample size **Limitations:** Single program, single specialty, ad-hoc measures	**12.4**
Unkart et al. (2016) [57]	Measure reduction in **general surgical** residency applications among candidates self-identified as “disadvantaged”	URM were: • Older at entry (24 v 23, *p* < .001) • Lower MCAT (30 v 33, *p* < .001) • More likely to choose a less competitive specialty (*p* < .03)	**URM/Gender:** No bias detected based on USMLE 1	**Strengths:** National cohort, multiple years intake, large sample **Limitations:** Aggregates heterogenous groups, limited follow-up	**12.4**
Villwock et al. (2019) [58] *Identified by specific search terms*	Measure effect of STAR tool for selecting **otolaryngology** residency candidates to interview	USMLE scores significantly increased after STAR tool No differences in gender/URM before/after introduction of STAR selection tool	**URM/Gender:** STAR selection tool did not increase representation of URM/Gender	**Strengths:** Moderate sample size **Limitations:** Single program, exploratory	**7.9**

*ARCP* Annual Review of Competence Progression, *CPST* Clinical Problem Solving Test, *DMG* Domestic Medical Graduate, *IMG* International Medical Graduate, *LoR* Letter of Recommendation, *PLAB* Professional and Linguistic Assessment Board, *SJT* Situational Judgement Test, *URM* Underrepresented minority

^a^ MERSQI scores include subscales which are not applicable for all articles; scores are scaled after removal of these subscales to allow comparison with a maximum score of 18 for all articles (Reed et al, 2007) [17]

### Under-represented minorities

Gender was by far the most frequently examined URM (22/35 articles: 62%), followed by international medical graduates (IMGs) (10/35: 28%). Nine articles reported multiple classes of URM (26%) and single articles considered age [36], personality [54], and geography [58] (each 3%).

### Methods used to investigate diversity of selection

Most of the studies were conducted in the US (27/35 articles; 77%) and after 2013 (24/35; 69%), with smaller contributions from the UK (7/35; 20%) and Canada (1/35; 3%). Surgery (18/35; 51%) and GP (5/35; 14%) generated the most articles of any single specialty, with most of the other specialties contributing one or no specific articles.

Table 3 summarises the strengths, limitations, and MERSQI scores of each article. The mean MERSQI score was 11.34 (SD: 2.61; range: 7.9–15.8) which is comparable with the previous literature using MERSQI as a measure of study quality. Across all articles, mean MERSQI scores were adequate for all domains except study design (1.25 out of 3) and data analysis (1.5 out of 3). The interrater reliability across all domains was in the fair (0.21–0.4) or moderate (0.41–0.6) range (Cohen’s Kappa) except where a lack of variation in the coded scores prevented calculation.

Consistent with the MERSQI scores of previous studies, closer examination of the collected articles revealed significant methodological limitations particularly in design and analysis (Table 3). Critically, a substantial minority only considered applicants that had already been selected into a training program, not those who were unsuccessful (26%). Prevalent limitations of the literature include that most of the articles were exploratory in nature (83%), and examined a single training program (56%), or a single specialty (78%).

Many articles had the strength of looking at a complete training cohort across a nation or state (34%), and most of the studies used large sample sizes (> 500 candidates; 69%). Across 35 articles, data was reported on 200,000 participants, with the UK articles averaging more than 17,000 participants and the US more than 2700 per article. Most of the studies also examined selection over multiple intake cycles (54% of articles considered more than 2 years of data). In contrast with the exploratory US literature, the 7 UK articles were part of a coordinated research effort using similar methods on national data sources focused on GP training and with a greater interest in the reliability of assessment of IMGs than other URMs.

While the methods, populations, and quality of the studies were too heterogeneous to allow meta-analysis, power was examined as a useful index of the quality of the research. Reflecting the primarily exploratory nature of the research, 17% of articles reported adequate power, 8% reported limited power, and 74% did not address power.

**Fig. 2:**
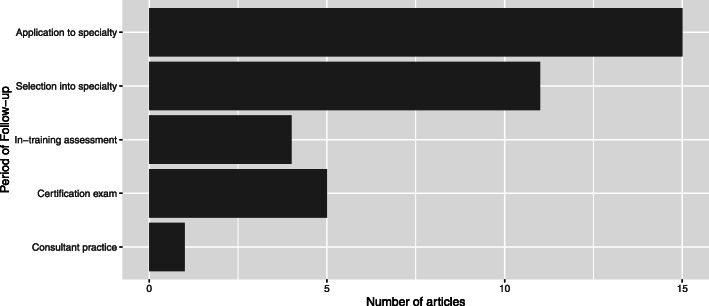
Length of follow-up

Also consistent with the exploratory nature of the research, most of the articles relied on retrospective cohort studies (89%), with only three prospective studies. Pre-selection academic achievement comprising MCQ exams were considered by most of the articles (74%), followed by letters of recommendation (33%), and a small number examining standardised or non-standardised interviews (8%) and selection centres (8%; sum greater than 100% as some studies looked at more than one selection method). Figure 2 shows that most of the literature had a limited follow-up period, with most articles considering only the process of application to training (15/35) or selection into a specialty (10/35). Few articles considered the impact of selection processes on in-training assessment (4/35) or certification exams (5/35), and only one looked at the effects of selection on consultant practice.

### Impact of pre-selection measures on diversity

Table 3 summarizes the impact of pre-selection measures on MSTS (authors claiming evidence of bias listed in bold). The lone Canadian article found no evidence of bias against IMGs. Three of seven UK articles concluded there was evidence of bias against URMs or IMGs. Eleven of twenty-seven US articles found evidence of bias, with two showing greater selection of women due to better performance on the USMLE 2 and interview; one showing lesser selection of women associated with worse performance on the USMLE 1; and five showing bias against women on letters of recommendation. None of the other significant results were supported across more than one study.

### Evidence that novel selection processes can increase diversity of selection

Two articles reported evidence on novel selection processes designed to increase diversity of selection. Gardner et al. [44] found that reducing the threshold of the USMLE 1 and adding a SJT with MCQs specifically designed for selection into surgical training increased the selection of URMs for interview by 8%. Villwock et al. [58] reported that an objective algorithm for selecting candidates for interview (Selection Tool for Applicants to Residency – STAR), designed to prevent unconscious bias with attention to multiple factors including geographical (eg candidates proximity to the selecting institution), did not increase the proportion of URMs offered interviews for otolaryngology training.

### Potential bias attributable to search strategy

Our replication of the basic search in the Scopus database did not identify any additional articles for review. Table 3 indicates which of the reviewed articles were identified by the addition of specific search terms to our basic search. Ten articles of the 35 reviewed were not retrieved by the basic search, of which 3 reported evidence of bias. The US literature provided 9 of the additional 10, with the other from the UK.

## Discussion

### Summary of findings and similarity to previous literature

The MSTS diversity literature focused mainly on under-selection of females into specialist training, followed by IMGs and then race or ethnicity. Apart from a small group of high quality studies from the UK with adequately powered large samples from national cohorts as part of the development of a systematic framework for GP trainee selection, evidence was limited by exploratory retrospective designs using convenience samples of single specialties and single training programs, with brief follow-up periods. Alongside the methodological limitations of the individual studies in this review, the large variations in the frameworks for MSTS between specialties within the same country, and even greater variations across countries, makes it difficult to draw confident conclusions from this literature. The results are consistent with recent reviews of medical school and specialty selection methods [14, 20] both in the dominance of US research with a smaller but more coherent set of articles from the UK; and with respect to their conclusions that reforming selection frameworks to achieve reliable and equitable selection will require research with greater methodological rigour, particularly longitudinal design and attention to validity.

Perhaps reflecting the relatively low diversity in surgical programs [14], half the studies examined one of the surgical subspecialties. Outside the GP focus of the UK literature, most non-surgical specialties were represented by a single article, or not represented at all. There was equivocal evidence of bias against the selection of females into specialist training, and contested evidence of bias against IMGs. The use of specific search terms in addition to the baseline search did not exclude any articles from review, but did identify an additional 10 articles, primarily from the US literature. The additional evidence reviewed appears unlikely to have significantly altered the analysis, conclusions, or recommendations of the review. Given the similar results of a recent review of MSTS not focused on diversity we believe our review is representative of the published literature.

### Methods used to investigate diversity in medical specialty selection

Although the methods used and populations sampled were diverse, almost all articles had retrospective cohort designs, and most of the research only followed up to the point of selection into training, with few looking as far as in-training assessments or certification exams. Durham et al. [39] is representative. They found that the USMLE 1 was the best predictor of selection into US neurosurgical training across all candidates. While reduced female selection was partially explained by lower USMLE 1 scores, multivariate analysis suggested that women were less likely to be selected even after controlling for the USMLE and other academic measures, which was interpreted as evidence of possible gender bias. This study shows two potential barriers to selection of female trainees: lower average USMLE 1 scores, which the authors implicitly accept as a reasonable index of ability; and gender bias of the whole selection process, which they do not consider acceptable.

It is notable that 26% of articles only reported data on people already selected into training. While these studies can compare URMs and others selected into training, it is difficult to explain barriers to MSTS without data about URMs who have been excluded from training.

Finally, while many studies noted that URM assessments before and during training are affected by multiple social, linguistic, and cultural factors, only one group of authors attempted to measure these systematically. Two studies showed that the training performance of IMGs in the UK were associated with their linguistic and cultural understanding [29] as well as their age, sex, level of experience, and socioeconomic status [30]. The complex interaction of selection measures, selection decisions, and broader social goals is well illustrated by these studies, which conclude that existing methods intended to ensure the equivalence of doctors trained outside the UK before entering specialty training may not be achieving that purpose. The authors speculate on whether tests of IMGs English fluency in the UK might in fact be measuring other cognitive constructs, and note their results imply that it would be necessary to significantly increase the cut-offs on IMG entrance exams for those exams to actually enforce equivalence between IMGs and domestic graduates. They suggest that due to the reliance of the UK health system on IMGs, such changes would risk severe workforce shortages, and consider alternatives that balance different social goals, such as increased support for IMGs, or other methods of testing [29].

### Evidence that assessments reduce specialty training diversity

Evidence on the impact of pre-training assessments on MSTS was interpreted in four main ways. Least problematic were studies which found no differences between URMs and other groups on pre-training assessments and selection into training or later outcomes and concluded there was no evidence of barriers to diversity caused by selection methods (Table 3, unshaded studies). The strength of this evidence is limited by the exploratory nature of most of the studies and the absence of power analyses.

A second group of studies found evidence that the selection of URMs into medical specialties was affected by specific biases in pre-selection measures, typically because low URM pre-selection scores were not consistent with equivalent in-training performance. The evidence included gender biases affecting letters of recommendation [41, 43, 47, 50, 59], sociolinguistic biases affecting selection interviews for IMGs [28, 30, 54], and bias against candidates sitting the USMLE 1 including women [15, 39] and IMGs [51]. This research focused on the need to measure and correct for biases, or to develop more valid alternative measures, which is also both reasonable and preliminary.

The final group of studies found that URMs had lower scores on pre-selection measures which were associated with a lower probability of selection and/or later outcomes. There were two quite different interpretations of these results. Some authors concluded that it is undesirable for low pre-selection scores to prevent URMs from entering training, even where they appear to accurately predict later performance, and suggested various ways of meliorating the impact such as relaxing cut-offs for URMs [40] or providing greater resources for IMGs [26]. Others concluded that the association of low pre-selection scores with lower scores on measures during training suggests that the under-representation is acceptable where it reflects lower levels of ability [26, 27, 29, 31].

The literature is not currently able to resolve these viewpoints. The view that URMs are under-represented because of ability rather than bias was most strongly asserted with reference to IMGs in the UK literature, while the view that pre-selection scores should not prevent URMs from entering specialty training was mainly associated with ethnicity and to a lesser extent gender in the US literature. The latter view raises the question whether there are selection methods that can facilitate URM entry into specialty training without unacceptable tradeoffs such as significantly reduced reliability of assessments.

### Evidence that novel selection methods can increase training diversity

Consistent with previous reviews of the impact on diversity of medical selection methods from medical school through consultancy we found that the diversity research is focused on academic pre-selection measures such as entrance or licensure exams, due to reliability, availability, and convenience, and that there is limited evidence of selection methods likely to increase training diversity [14, 20, 60]. Even critics of non-specific academic pre-selection measures acknowledge that there is a need for some method of short-listing applicants for medical specialty training programs due to the highly competitive nature of a system where as many as 800 applications might be received for 5 positions on a general surgery program [17]. As a result, novel methods of selection must either replace existing reliable measures, or augment/modify them in some way.

Of two studies reporting on efforts to increase diversity of medical training by increasing the selection of URMs into training, one claimed success [44] and one did not [58]. The study claiming success did not replace the USMLE as an initial screen, but rather added a specially designed second screening tool with unreported psychometric properties. Given the main reason the USMLE 1 has been almost universally used as a specialty screen in the US is because it is highly reliable and does not require additional resources, it is unclear whether the extra resources and reduced reliability of this approach is justified by an 8% increase in URM interviews.

We did not discover any evidence suggesting that diversity can be increased by using existing measures in a different way, for example by changing the relative weight given to the various measures and methods described in Table 1.

### Lessons for global health systems

The literature provides preliminary evidence requiring replication that existing measures used for MSTS may be biased against women and IMGs in specific circumstances, and one article which showed it is possible to increase the number of URM interviews, if not the number of URMs entering training, by screening for specific characteristics. Limited reporting of statistical power leaves open the possibility that material biases against URMs exist but have not been adequately tested. Some authors concluded that the poor performance of IMGs on assessments from selection through to certification were reliable indicators of ability, although a more nuanced view was that the main issue is unequal access to cultural and linguistic resources, remediable by adequate support and training [28].

Despite these limited results, and the absence of research outside the US and UK, the present review is relevant to other countries looking to reform their MSTS frameworks to improve diversity, particularly in the context of significant recent developments. In the US, the Federation of State Medical Boards (FSMB) and National Board of Medical Examiners (NBME) have decided to change reporting of the USMLE 1 to pass/fail rather than graded, preventing its use as a MSTS instrument [61, 62]; and the University of California and other US institutions have decided to eliminate MCQ entrance exams [63]. These changes were presented as efforts to address barriers that directly contribute to the under-representation of some groups in higher education generally and medical specialist training in particular, and both highlight the relative tension between reliability and validity discussed above [14, 20]. In effect, these US-based institutions have decided that the advantages of reliable assessments, which primarily benefit privileged groups, are outweighed by the disadvantages of limited validity, which tend to directly disadvantage less privileged groups, and indirectly broader society.

At the same time that use of the most common standardised MSTS instrument in the US is being prevented, the UK has moved towards greater reliance upon standardised testing, with multiple medical colleges in the UK adopting the Multi-Specialty Recruitment Assessment (MSRA) tool [64, 65]. While the evidence base is limited (for example, a PubMed search for “Multi-Specialty Recruitment Assessment” on 20.03.21 returned only 1 relevant article, a letter published in 2021), the MSRA seeks to find a better balance between reliability and validity by developing multiple sources of evidence and reducing the influence of more subjective selection methods [30]. It includes computer-based tests, including SJTs and CPSTs, which have been suggested to be relatively more valid than other measures used for medical selection [20]. It is interesting that uptake and weighting of the MSRA in selection decisions by UK medical colleges appears to have been accelerated by covid, due to the reduced social contact required by computer-based testing versus other methods like interviews [66].

We do not propose to explore the complex broader social context which will have influenced these contrasting developments in the US and UK, other than noting the preoccupation with equity in both countries represented by movements such as Black Lives Matter [67] and #MeToo [68]; and the UK’s exit from the European Union which has been linked with immigration patterns and the desire for increased quality of health care [69]. However, we suspect such factors may have played a part in the divergent paths of the US and UK with respect to MSTS, with the US relatively prioritising equity over reliability; and the UK relatively prioritising reliability while trying to improve the validity of MSTS by systematically drawing on multiple sources of evidence.

The limitations of the reviewed literature make it difficult to predict the impact of changes in MSTS frameworks intended to increase diversity. The US and UK examples suggest that other countries considering reforming their MSTS frameworks might be tempted to prioritise the reliability of pre-existing academic exams modelled on the UK, over the uncertainty associated with the US approach, however justifiable as a means of improving diversity. It is too early to judge the results of either approach. As a result, the only sure recommendation from this literature for countries hoping to improve the reliability of MSTS and increase diversity is the need to closely monitor the impact of changes to avoid or respond rapidly to unintended consequences. In the absence of evidence of reliable selection methods that increase diversity, moving away from existing MSTS measures may leave URMs worse off [44], particularly if specialty programs revert to methods such as alumni networks, letters of recommendation, or other techniques that are biased towards those with greater resources. While acknowledging the trade-offs between the interests of patients, minorities, and society in general, some have argued that this lack of evidence justifies selection into medical training by a weighted lottery as the only existing method likely to be effective in achieving truly equitable levels of diversity in medical workforces [70].

Achieving increased diversity by more reliable methods than a weighted lottery will require two main advances in the literature. Current MSTS frameworks rely on pre-selection academic results rather than measures specific to specialties, alongside more subjective methods such as letters of recommendation, interview, and references. The only specialty specific measures identified in this review were for GP training (UK) [28] and a single surgical training program (US) [44]. It has been argued that the use of general measures for specialty selection has led to an arms race with constantly escalating scores required for entry [17]. Developing more specific measures may allow URMs to focus on targeted knowledge and skills and to benefit from reduced competition for places. There is likely to be a trade-off between greater validity and reduced reliability for such measures given the much larger number of people who take entrance exams for medical school and licensure for medical practice than enter any medical specialty. The limited evidence available for the MSRA, adapted from the specific measures developed for GP selection [28], makes it difficult to anticipate what impact its adoption by other medical colleges will have on the diversity of their workforces.

Second, in order to resolve whether under-representation in medical specialties is due to biased measures, differential ability, or other factors such as distribution of resources, it will be necessary to complete adequately powered prospective studies with successful and unsuccessful applicants, comparing general exam measures with specialty specific measures and accounting for the effect of confounding factors such as age, linguistic ability, cultural knowledge, and economic status. Well-designed research should generate results that are somewhat generalisable between countries, but local conditions will always be relevant. This type of study would also help identify what support measures might be necessary to improve diversity, assuming that differential performance at the point of selection is due to unequal resources rather than differential capacity.

### Strengths and limitations

The review involved systematic searches of multiple databases supported by hand-search and reference-tracking, and comparison of literature from the US, UK, and Canada, with article quality evaluated using the MERSQI. It was limited by the absence of meta-analytic statistics due to the heterogeneity of the studies. Confident conclusions were limited by the exploratory nature of most of the literature, the absence of replications, and retrospective/convenience-based designs. The possibility of bias in the search strategy and/or results was explored and quantified, but cannot be entirely ruled out, although observed imbalances results were similar to a previous review with a broader focus. This is the first review to examine the impact of MSTS methods on medical workforce diversity, which is an issue of immediate interest in the context of a divergence in the US/UK use of standardised tests that may provide guidance for other countries looking to reform MSTS.

## Conclusions

Consistent with the broader medical selection literature, a focused review of the impact of MSTS methods on the diversity of medical specialist workforces suggests those actually responsible for selection decisions continue to value the reliability of pre-selection academic results, with little evidence that this is a significant cause of the under-representation of some groups, albeit the evidence base is small, underpowered, and focused almost entirely on the US and UK. Some stakeholders have prioritised alternative social goals including assessment validity and workforce diversity. In the context of strong cultural movements addressing perceived inequities, MSTS frameworks in the US and UK are moving in different directions, with the US reducing reliance on standardised measures to promote diversity, and UK medical colleges increasing their use but attempting to improve validity by drawing on multiple sources of evidence. The fact that the two most researched MSTS frameworks are taking different paths on an uncertain evidence base demonstrates both the strong extra-scientific pressures, and the need for rigorous international longitudinal research on causes of under-representation of minorities and effective means to answer these. Countries considering MSTS reform to achieve socially accountable health systems with appropriately diverse health workforces must support systematic research in their own training systems, and monitor for and respond to unanticipated consequences of change.

## Data Availability

Not applicable.
